# The influence of gender on inheritance of exceptional longevity

**DOI:** 10.18632/aging.100763

**Published:** 2015-06-22

**Authors:** Jennifer A. Deluty, Gil Atzmon, Jill Crandall, Nir Barzilai, Sofiya Milman

**Affiliations:** ^1^ Albert Einstein College of Medicine, Bronx, NY 10461, USA; ^2^ Department of Medicine, Division of Endocrinology, Albert Einstein College of Medicine, Bronx, NY 10461, USA; ^3^ Institute for Aging Research, Albert Einstein College of Medicine, Bronx, NY 10461, USA; ^4^ Department of Genetics, Albert Einstein College of Medicine, Bronx, NY 10461, USA; ^5^ Department of Biology and Human Biology, University of Haifa, Haifa, Israel

**Keywords:** centenarians, longevity, gender, inheritance

## Abstract

While the search for genetic contributors to exceptional longevity has yielded candidates, gender differences in inheritance have generally not been considered. The aim of this study was to investigate gender specific differences in the inheritance of exceptional longevity. Using a standardized questionnaire, we assessed the parental ages of death of Ashkenazi Jews with exceptional longevity and their spouses without exceptional longevity, who served as controls (n=1,114). Mothers of centenarian males and females had significantly longer lifespans compared to the mothers of non‐ centenarians, 79.0 ± 13.4 vs. 73.0 ± 16.3 years, p<0.01 and 75.7 ± 15.8 vs. 70.5 ± 18.0 years, p=0.02, respectively. There was also a trend toward longer lifespan among the fathers of centenarian men compared to the lifespan of fathers of non‐ centenarian men, 73.5 ± 17.0 vs. 69.5 ±15.0 years, p=0.07. The lifespan did not differ between the fathers of centenarian and non‐centenarian daughters. Logistic regression models revealed that the odds of being a centenarian for the female and male offspring increased by 21% and 31%, respectively, for every additional 10 years of life achieved by the mother (p<0.05). These findings support a gender‐specific inheritance pattern of human longevity and may help focus the search for longevity genes.

## INTRODUCTION

It is well established that human longevity is at least in part genetically determined, with an estimated heritability between parents and offspring on the order of 0.20 to 0.30 [1–3]. This genetic influence becomes even more pronounced as individuals achieve older age, with the heritability increasing substantially for each 10 year increment in the cohort's age of death [4]. The effect of inheritance on lifespan seems most apparent in the centenarian and supercentenarian populations [5, 6]. These observations have generated many attempts to identify the genes and inheritance patterns responsible for exceptional longevity in different populations [6, 7]. Utilizing a variety of genetic approaches, such as the candidate gene approach and Genome Wide Association Studies (GWAS), researchers have tested numerous hypotheses that focus on the inheritance of exceptional longevity, including inheritance of mitochondrial genes [8], somatic genes [6, 8], and x-linked genes [2, 9]. Yet, little is known regarding whether the inheritance of longevity is influenced by the gender of the parent. In this study, we aim to investigate the gender specific differences in the inheritance of longevity in a cohort of Ashkenazi Jews who have achieved exceptional longevity in an attempt to better understand the inheritance patterns and localize longevity genes.

## RESULTS

### Parental Ages of Death

**Figure 1:**
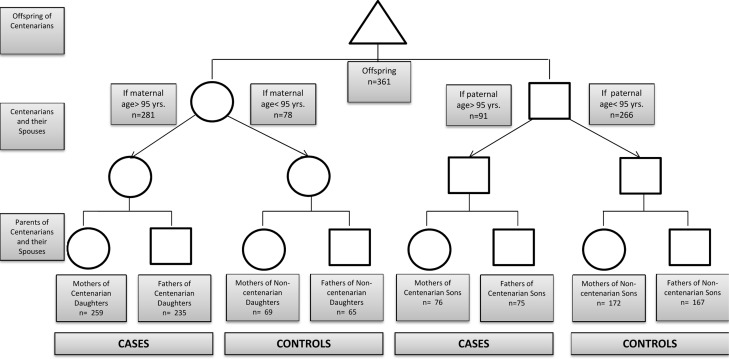
Experimental design Offspring provided information about their parents and grandparents. If the parent was > 95 years of age (centenarian), the grandparents of that lineage were deemed as cases. If the parent was <95 years of age (non‐centenarian), the grandparents of that lineage were deemed as controls.

Data from 361 offspring questionnaires was included in this analysis. Figure 1 provides a detailed description of the study cohort. Maternal age of death was available for 333 centenarians and for 241 of their non-centenarian spouses (Figure 1). Paternal age of death was reported for 308 centenarians and for 232 of their non-centenarian spouses. For 291 centenarians and 219 non-centenarians the age of death for both parents was known. The median age (interquartile range) of female centenarians (n=281) was 97 years (96-99 years), range 95-108 years, and the median age of male centenarians (n=91) was 97 years (95-99 years), range 95-105 years. The median lifespan of female spouses of centenarians (n=78) was 85 years (77-90 years), range 42-94 years and of male spouses of centenarians (n=266) was 75 years (66-84 years), range 41-94 years.

**Figure 2:**
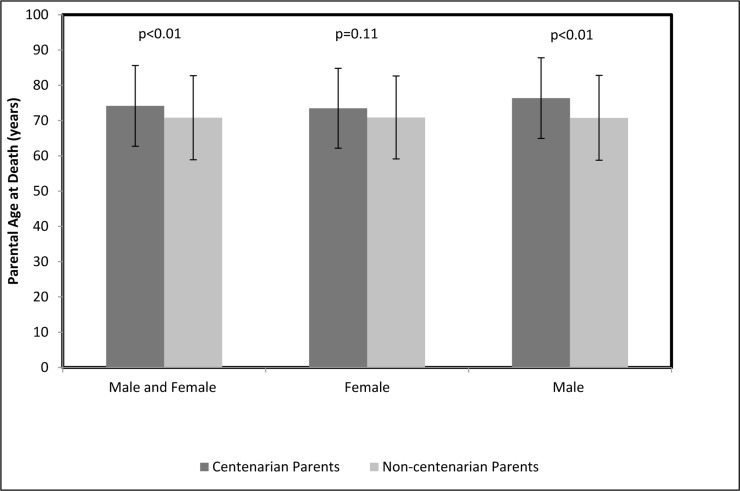
Parental age at death Comparison between the average parental ages at death among centenarians and non‐centenarians for both genders of centenarians combined, females only and males only.

Age of death of both parents was reported for 224 female centenarians, 63 female non-centenarians, 69 male centenarians and 156 male non-centenarians. The average parental age of death was significantly higher among the parents of the centenarian cohort than among the parents of their control counterparts, 74.2 ± 11.4 years and 70.8 ± 11.9 years, respectively, p<0.01 (Figure 2). After stratifying the centenarian and control cohorts by gender, this trend persisted only among the males, with the mean parental age of death of male centenarians being 76.4 ± 11.4 years and of male non-centenarians being 70.8 ± 12 years, p<0.01 (Figure 2). An analysis that compared the mean age of death of the parents of female centenarians and female non-centenarians did not find significant differences, with the mean parental lifespan being 73.5 ± 11.5 years and 70.9 ± 11.7 years, p=0.11, respectively (Figure 2).

**Figure 3:**
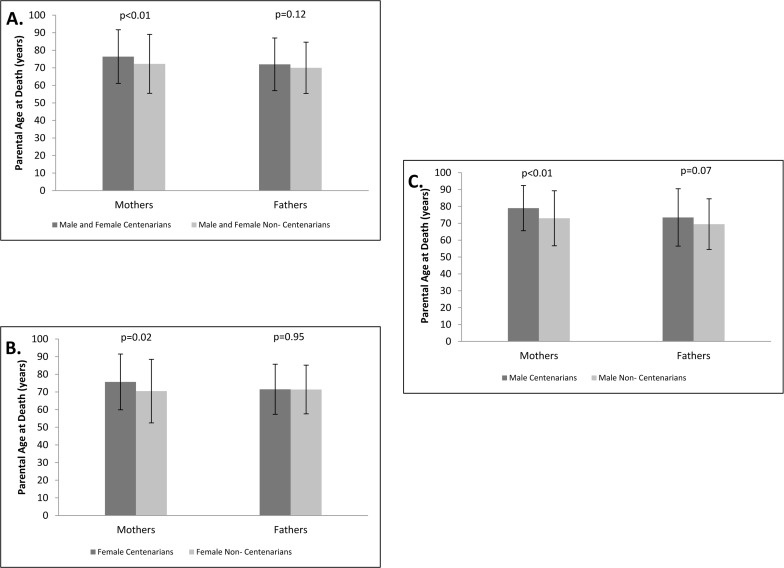
Maternal and paternal age at death Maternal and paternal ages at death among the parents of centenarians and non‐centenarians. **A.** Both genders of centenarians combined. **B**. Females only. **C.** Males only.

An analysis that stratified the parents by gender demonstrated that the mothers of centenarians had longer average lifespan compared to the mothers of non-centenarians, with the mean age at death of the mothers of centenarians being 76.4 ± 15.3 years and the mean age at death of the mothers of non-centenarians being 72.3 ± 16.8 years, p<0.01 (Figure 3A). The average lifespan achieved by the fathers of centenarians was 72.0 ± 15.0 years compared to 70.0 ± 14.6 years achieved by fathers off non-centenarians, p=0.12 (Figure 3A).

Further gender stratification of the centenarian and control cohorts revealed that the average age at death among the mothers of centenarian women (n=259) was 75.7 ± 15.8 years compared to 70.5 ± 18.0 years among the mothers of non-centenarian women (n=69), p=0.02 (Figure 3B). Fathers of centenarian daughters (n=235) had an average life span of 71.5 ± 14.2 years, while the fathers of non-centenarian daughters (n=65) lived, on average, 71.4 ± 13.8 years, p=0.95 (Figure 3B). The mean ages of death of the mothers of centenarian men (n=76) versus the mothers of non-centenarian men (n=172) were 79.0 ± 13.4 years vs. 73.0 ± 16.3 years, respectively, p<0.01 (Figure 3C). The fathers of centenarian sons (n=75) achieved an average lifespan of 73.5 ± 17.0 years, whereas the fathers of non-centenarian sons (n=167) had a mean lifespan of 69.5 ±15.0 years, p=0.07 (Figure 3C).

### Logistic Regression Models

The logistic regression models demonstrated that the odds ratio (95% confidence interval) of a daughter being a centenarian was 1.23 (0.96-1.56), p=0.09, and of a son being a centenarian was 1.52 (1.17-1.98), p<0.01, per every additional 10 years in average lifespan achieved by the parents. The results of modeling maternal vs. paternal associations revealed that the OR of a daughter being a centenarian was 1.21 (1.03-1.41), p=0.02, for every 10 year extension in the lifespan of the mother. However, this relationship was not noted between daughters and their fathers, OR 1.01 (0.83-1.23), p=0.92. The OR of a son being a centenarian was 1.31 (1.08-1.59), p<0.01, for every 10 year extension in the lifespan of the mother and 1.18 (0.99-1.42), p=0.07, for every 10 year extension in the lifespan of the father.

The odds ratio of a male being a centenarian was 1.8 (1.1-3.1), p=0.03, if the mother reached at least 80 years of age and 2.0 (1.1-3.5), p=0.02, if the father reached at least 80 years of age. If the combined average lifespan of both parents was at least 80 years, then the odds of the son being a centenarian were even greater, OR 2.3 (1.2-4.1), p<0.01. The odds of a female being a centenarian if her mother achieved a lifespan of at least 80 years were 1.6 (0.9-2.8), p=0.10, which trended toward significance. However, no similar association was noted for females whose fathers achieved age 80 or older, OR 1.1 (0.6-2.1), p=0.68. Similarly, there was no significant association between the daughters being centenarians and the combined average lifespan of both parents, OR 1.5 (0.8-2.8), p=0.22.

## DISCUSSION

This study demonstrated that mothers of centenarian males and females had significantly longer lifespans compared to the mothers of non-centenarians. There was a trend toward longer lifespan among the fathers of centenarian men compared to the lifespan of fathers of non-centenarian men, but interestingly the lifespan did not differ between the fathers of centenarian and non-centenarian daughters. Furthermore, the odds of being a female or a male centenarian increased by 21% and 30%, respectively, for every additional 10 years of life achieved by their mother. The odds were even greater for males whose parents in combination exhibited extended average lifespan, with their odds of being centenarians increasing by 52% per every additional 10 years in lifespan achieved by their parents. Our findings suggest that male centenarians may inherit their longevity from both maternal and paternal lineages, whereas among the female centenarians only maternal lineage appears to have an effect on the inheritance of longevity. This may have implications for a model of gender specific inheritance patterns of human longevity genes. Furthermore, these findings may help direct the search for longevity genes in different areas of the genome depending on the subjects’ gender, such as mitochondrial DNA or sex chromosomes.

The differences in maternal versus paternal inheritance cannot be attributed simply to the fact that females have longer lifespans. Though current epidemiologic data demonstrates that females have longer lifespans compared to their male counterparts, this gender discrepancy in lifespan is not applicable to the parents of the centenarians and non-centenarians born in the 1850's, who were the subjects of this investigation. Demographic records indicate similar life spans among males and females of that birth cohort, both in the United States and in Europe [10]. Furthermore, comparing mothers of centenarians to mothers of non-centenarians and similarly fathers of centenarians to fathers of non-centenarians in the same cohort of individuals removes any bias that may arise due to differences in lifespan of each gender. Thus, we believe that the identified age differences among the mothers of centenarians and non-centenarians represent true differences in lifespan irrespective of inherent gender-specific aging.

It should also be noted that the lifespan among our controls, who exhibited average maternal lifespan of 72.3 years and an average paternal lifespan of 70.0 years, is significantly higher than that previously found for this cohort born in the 1850's. The average documented lifespan for individual born in the 1850's and who reached at least age 20, thereby achieving reproductive age, ranged from 58 to 63 years in the literature [10, 11]. Thus, the magnitude of inheritance as found in this study may be an underestimation of the true value.

The association between extended lifespan and inheritance of maternal genes may suggest a mode of mitochondrial gene inheritance. Mitochondrial regulation of reactive oxidative species and its role in DNA damage makes it a primary target for aging related research. A recent study demonstrated mutations in the OXPHOS complex I in subjects who reached 90 years of age or older, supporting a beneficial role for this complex in aging [8]. Other mutations in complex III and V have been shown to be detrimental to the aging process [8]. Though the allelic composition of the mitochondrial DNA of the Ashkenazi cohort is similar to that of the non-Jewish Caucasian population [12], more work needs to be done to further elucidate the role of mitochondrial gene inheritance in human longevity.

The apparent lack of longevity transmission from fathers to daughters in contrast to fathers to sons supports the possibility of a Y-chromosome or male component transmission. This suggests gender specific modes of genetic transmission and the possibility of an even greater transmissibility in males over females. Additionally, the genomic influence on longevity that is exerted by both parents in combination, as appears to be the case in males, may be particularly important for the transmission of recessive genes. Several homozygous recessive longevity genotypes have been identified, including the *CETP* and *APOC3,* with the gender affects on these genes not fully explored yet [13, 14]. This divide of gender specific transmission lays the groundwork to answer the question of the molecular modes of gender specific transmission.

Longer parental lifespan has been implicated in protection of the offspring from numerous diseases. In fact, not only the offspring of centenarians appear to have this protection [15, 16], but also do the offspring of parents who have achieved less extreme lifespans. Offspring of parents who lived to at least age 85 years demonstrated a 43% lower incidence of Alzheimer's disease compared to the offspring of parents who did not reach age 85 [17]. Similarly, in the Framingham study, parental lifespan of 85 years or older was associated with lower blood pressure and Framingham risk score progression in the offspring [18]. In the Diabetes Prevention Program, age 80 or older in the parents was related to about 30% lower hazard in developing diabetes mellitus type 2 [19]. This advantage in survival may be genetically transmitted to the offspring and may be amplified in the future generations, as the average lifespan continues to lengthen in the general population.

Our findings of gender-specific modes of inheritance in an Ashkenazi Jewish population of exceptional longevity are supported by data from another genetically unique cohort of Icelanders, who were studied as part of the Decode Iceland Genealogy Registry [20]. In the Islandic population, sons and daughters of long lived mothers, as well as sons of long lived fathers (>90 years), had a 25% lower age specific death rate than the sons and daughters of the control group. In contrast, female offspring of long lived fathers showed only a 14% decrease in death rate compared to the control group. Demographic data from the United States also supports gender-specific influences on longevity [21], revealing that centenarian male gender is significantly associated with the lifespan of other male relatives but not female relatives. The consistency of results between populations may help to widen the generalizability of these results to aging populations at large.

The limitations of our study include a relatively small sample size, particularly for male centenarians. The lack of statistical power likely contributed to the fact that we were only able to document trends but not statistically significant relationships between the lifespans of male offspring and their fathers. The format of survey and the cross sectional study design may lead to general recall bias. However, since the data for both the cases and controls was provided by the same individuals there is no concern that the recall bias was differential and therefore we do not suspect that it would bias results in one direction or another. The fact that our study was conducted in a genetically homogenous population of Ashkenazi Jews, with controls and cases derived from the same families and birth cohorts, is a significant strength of our study.

Although our findings support the association between gender and genomic inheritance of longevity, gender-specific environmental influences on longevity have not been completely ruled out. Other investigators suggested that the observed discrepancy in the inheritance of lifespan between fathers and sons versus fathers and daughters may be explained by the fact that in the past occupation was usually passed down from the father to the male children [21, 22]. Males with occupations that were considered to be advantageous for survival, such as farming [22], were more likely to be centenarians. Females, on the other hand, who were more likely to share their environmental exposures with their husbands rather than their male blood relatives would not benefit from these advantageous environmental factors that may have been beneficial for their male siblings.

In conclusion, we identified that in males both maternal and paternal inheritance are likely contributors to exceptional longevity, whereas among females maternal inheritance appears to be more influential. Several gender-specific genomic mechanisms may be at play in influencing exceptional longevity, such as maternally or paternally inherited gene variants, mitochondria derived genes, or a combination of these components. These finding lay the groundwork for the identification of longevity genes whose inheritance is gender-dependent.

## METHODS

### Study participants

Ashkenazi Jewish individuals with exceptional longevity (centenarians), characterized by living independently at age 95 years, which was considered a reflection of good health, were recruited from the Northeastern United States for the Longevity Genes Project at Albert Einstein College of Medicine beginning in 1998, as previously described [15]. As part of this study, the offspring of centenarians were also recruited and completed a structured questionnaire that included information about the ages of death of their parents (centenarians and their spouses), as well as the ages of death of their grandparents (centenarians’ and their spouses’ parents). The parents of centenarians were deemed as cases (Figure 1). The parents of spouses were considered controls, provided that the spouse did not achieve age 95 or greater (Figure 1). Seven couples were identified where both partners were centenarians. The use of the questionnaire completed by the offspring to ascertain the ages of both case and control groups was beneficial to reduce recall bias and ensure similar environmental factors within the family units.

Data from 361 offspring questionnaires was included in this analysis. Sibling offspring were excluded so that only one report from each family was represented in our data. Ages were validated by using reports of multiple centenarian offspring when available. In the event that multiple siblings reported about the same families and no discrepancies existed between offspring accounts, only the most detailed account of parental and grandparent ages was included. If they were exactly the same, one was chosen to represent the family. When discrepancies arose over the ages between each of the centenarian offspring account, data was confirmed by comparing it to the reported age of this parent as reported by the centenarian themselves. If this was not available and to resolve reported age discrepancies of the control parents, where there was no additional data to confirm ages, an average of the ages as reported by each offspring of centenarians was obtained (n=26). When ages were recorded as “70+” or “70's”, they were uniformly assumed to be 70. Ages documented in the record as “early 70” and “late 70” were transcribed in the dataset as 72 and 78, respectively. A reported age of “mid 70” was consistently assumed to be 75. Statements like “believe 90's” and “90?” were rarely found and were recorded consistently as 90. The mean parental lifespan was calculated only for those individuals for whom both maternal and paternal age of death information was available, by taking an average of the maternal and paternal age at death.

### Statistical analysis

Statistical analysis was conducted using STATA software version 12 (StataCorp LP, College Station, TX). Ages were compared using bivariate statistics, with non-parametric tests used when appropriate. Normality was assessed by observation of the histograms. Results for the logistic regression models are reported as odds ratios (OR) with 95% confidence intervals (CI). A p-value <0.05 is considered statistically significant.
